# TRIB2 regulates normal and stress-induced thymocyte proliferation

**DOI:** 10.1038/celldisc.2015.50

**Published:** 2016-03-15

**Authors:** Kai Ling Liang, Caitriona O’Connor, J Pedro Veiga, Tommie V McCarthy, Karen Keeshan

**Affiliations:** 1 Paul O’Gorman Leukemia Research Centre, Institute of Cancer Sciences, College of Medical, Veterinary and Life Sciences, University of Glasgow, Glasgow, UK; 2 School of Biochemistry and Cell Biology, University College Cork, Cork, Ireland

**Keywords:** cell cycle, genotoxic stress, oncogenic stress, proliferation, pseudokinase

## Abstract

TRIB2, a serine/threonine pseudokinase identified as an oncogene, is expressed at high levels in the T-cell compartment of hematopoiesis. The proliferation of developing thymocytes is tightly controlled to prevent leukemic transformation of T cells. Here we examine *Trib2* loss in murine hematopoiesis under steady state and proliferative stress conditions, including genotoxic and oncogenic stress. *Trib2*^*−/−*^ developing thymocytes show increased proliferation, and *Trib2*^*−/−*^ mice have significantly higher thymic cellularity at steady state. During stress hematopoiesis, *Trib2*^*−/−*^ developing thymocytes undergo accelerated proliferation and demonstrate hypersensitivity to 5-fluorouracil (5-FU)-induced cell death. Despite the increased cell death post 5-FU-induced proliferative stress, *Trib2*^*−/−*^ mice exhibit accelerated thymopoietic recovery post treatment due to increased cell division kinetics of developing thymocytes. The increased proliferation in *Trib2*^*−/−*^ thymocytes was exacerbated under oncogenic stress. In an experimental murine T-cell acute lymphoblastic leukemia (T-ALL) model, *Trib2*^*−/−*^ mice had reduced latency *in vivo*, which associated with impaired MAP kinase (MAPK) activation. High and low expression levels of *Trib2* correlate with immature and mature subtypes of human T-ALL, respectively, and associate with MAPK. Thus, TRIB2 emerges as a novel regulator of thymocyte cellular proliferation, important for the thymopoietic response to genotoxic and oncogenic stress, and possessing tumor suppressor function.

## Introduction

TRIB2 is a member of the mammalian Tribbles family of serine/threonine pseudokinases (TRIB1–3). Studies focused on the pathological role of TRIB2 in various disease states, including hematological malignancy, solid tumors, autoimmune and inflammatory diseases, have identified TRIB2 as a critical signaling modulator and mediator [1]. However, it is unclear if this is true in a physiological context where regulation of diverse signaling pathways is cell type and developmental stage dependent. Studies of Tribbles orthologues in Xenopus [2] and Drosophila [3–5] highlight an evolutionary conserved role for Tribbles in the regulation of normal cellular proliferation. In these organisms, Tribbles coordinates cell division and morphogenesis to ensure proper organ development. In Drosophila, Tribbles ensuring mitosis occurs in a timely manner by regulating String/CDC25 turnover at protein level [3–5]. Histological study of a *Trib2* knockout mouse model showed no phenotypic defects in various organs during embryogenesis [6]. These data suggest TRIB2 function is required for proper cellular behavior rather than tissue organization.

In hematological malignancies, TRIB2 has been implicated in acute myeloid leukemia (AML), as well as T-cell acute lymphoblastic leukemia (T-ALL). Enforced expression of *Trib2* by retroviral transduction induces potent murine AML [7] and *Trib2* is a target gene of MEIS1 [8] in HOX-induced murine leukemia. In human AML, elevated expression of *Trib2* was shown to be driven by the transcription factors E2F1 [9] and NOTCH1 [10] where the latter was found to be aberrantly activated in an AML subset that has a mixed myeloid/T-lymphoid phenotype [10]. In human T-ALL, high *Trib2* expression was found to be associated with activated NOTCH1 signaling [11]. Indeed, *Trib2* was also identified as a downstream target of PITX1 [12] and TAL1 [13], transcription factors that are also aberrantly expressed in T-ALL. These oncogenic transcription factors involved in TRIB2 regulation and associated AML and T-ALL are important players in lineage specification during normal hematopoiesis [14–18], suggesting a potential role for TRIB2 in normal hematopoiesis.

We previously showed that *Trib2* expression is highest in the T-cell lineage in normal hematopoiesis and is regulated during αβ T-cell development in the thymus [19]. The thymus is a lymphoid organ where progenitors from the bone marrow (BM) commit to T-cell lineage development, differentiate into functional naive T cells, and are then exported to the periphery as part of the adaptive immune system. Here we show that TRIB2 is a novel cell cycle brake essential for balanced proliferation of developing murine thymocytes. Loss of TRIB2 causes developing thymocytes to be highly proliferative but with heightened sensitivity to *in vivo* genotoxic drug treatment. As such, *Trib2* ablation accelerates thymopoietic recovery following genotoxic insult. However, in cells expressing a T-cell oncogene, the absence of TRIB2 led to enhanced T-cell leukemic transformation associated with impaired MAP kinase (MAPK) signaling. In the human disease, low levels of *Trib2* expression correlate with a mature T-ALL phenotype and high levels of expression with an immature phenotype. In accordance with our experimental model, MAPK signaling correlates with *Trib2* expression in T-ALL.

## Results

### TRIB2 is dispensable for murine hematopoiesis in the bone marrow

**Figure 1:**
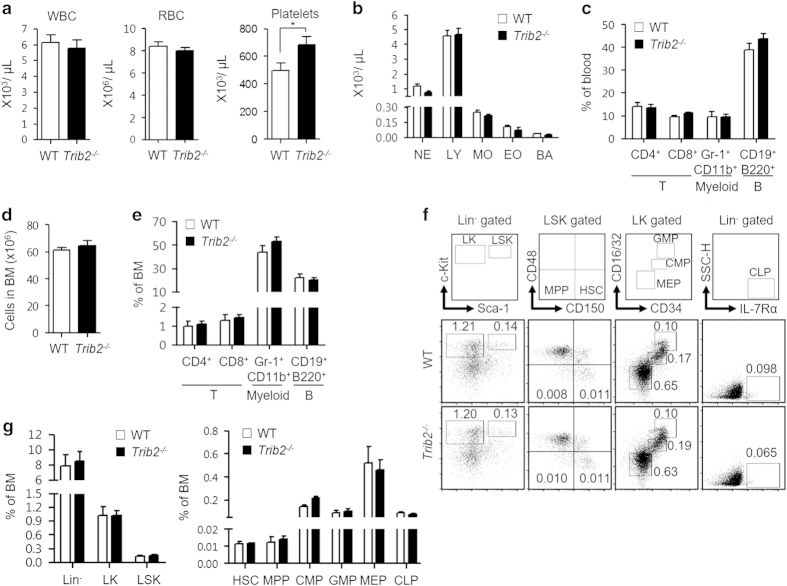
TRIB2 loss does not affect murine hematopoiesis in bone marrow. Complete blood counts (**a**) and WBC differential counts (**b**) of WT (*n*=13) and *Trib2*^*−/−*^ (*n*=22) mice were determined by hematology analyzer. BA, basophils; EO, eosinophils; LY, lymphocytes; MO, monocytes; NE, neutrophils. (**c**) The distribution of mature myeloid, B and T-cells in the blood of WT (*n*=3) and *Trib2*^*−/−*^ (*n*=10) mice were measured by flow cytometry using the indicated lineage specific cell surface markers. (**d**) BM cellularity (*n*=5–7 per genotype) was counted by trypan blue exclusion after RBC lysis of the cell suspension collected from two pelvises, femurs and tibias. (**e**) The distribution of myeloid, B and circulating T-cells in the BM of WT (*n*=3) and *Trib2*^*−/−*^ (*n*=9) mice. (**f**) Immunophenotyping of HSPCs populations (HSC, MPP, CMP, GMP, MEP and CLP) in BM. Each sub-population is indicated in the outlined areas (top row). The corresponding values in the representative staining profile of WT (middle row) and *Trib2*^*−/−*^ (bottom row) mice (*n*=3 per genotype) are frequency of BM and graphed in **g**. CLP, Lin^−^IL-7Rα^+^; CMP, LK CD34^+^CD16/32^lo^; GMP, LK CD34^+^CD16/32^hi^; HSC, LSK CD150^+^CD48^−^; Lin, lineage; LK, Lin^−^c-Kit^+^; LSK, Lin^−^Sca-1^+^c-Kit^+^; MPP, LSK CD150^−^CD48^−^; MEP, LK CD34^−^CD16/32^−^; SSC-H, side scatter-height. For **a**, unpaired Student’s *t*-test was used for statistical analysis. *P<0.05, all quantified data are presented as mean and s.e.m. CMP, common myeloid progenitor; CLP, common lymphoid progenitor; GMP, granulocyte-macrophage progenitor; MEP, megakaryocyte-erythroid progenitor; MPP, multipotent progenitor; RBC, red blood cell; WBC, white blood cell.

To investigate whether TRIB2 has a role in normal hematopoiesis, we examined the hematopoietic system of a *Trib2* knockout mouse model (129S5-*Trib2*^*tm1Lex*^, referred as *Trib2*^*−/−*^ hereafter) where the coding and noncoding regions of exon 1 were disrupted and thus enabled *Trib2* genotyping by PCR analysis (Supplementary Figure S1; Supplementary Table S1). Compared with wild-type (WT) mice, *Trib2*^*−/−*^ mice had similar red blood cell and white blood cell differential counts, except for the platelet count which was significantly higher but within the normal physiological range (Figure 1a and b). In addition, no difference was found between WT and *Trib2*^*−/−*^ mice in the distribution of mature myeloid, B and T-cells in the blood (Figure 1c). These data indicate that loss of TRIB2 does not affect terminal differentiation and production of mature blood cells of different lineages. We next studied the BM, the primary site of adult hematopoiesis, to determine whether TRIB2 influences hematopoietic cell fate choice at an earlier stage. *Trib2*^*−/−*^ mice had similar BM cellularity compared with WT mice (Figure 1d). No difference was found in the frequency of circulating CD4^+^ and CD8^+^ T-cells, lineage committed Gr-1^+^/CD11b^+^ myeloid cells and CD19^+^/B220^+^ B-lymphoid cells (Figure 1e). Moving up the hematopoietic hierarchy, *Trib2*^*−/−*^ mice had similar frequencies of hematopoietic stem and progenitor cells (HSPCs), including multipotent progenitors, common lymphoid progenitors, granulocyte-macrophage progenitors, megakaryocyte-erythroid progenitors, common lymphoid progenitors and hematopoietic stem cells (HSCs) compared with WT mice (Figure 1f and g). Hence, TRIB2 is not essential for hematopoietic cell fate choice as loss of TRIB2 did not lead to skewing of hematopoietic cell differentiation. Thus, at steady state, *Trib2* ablation does not affect the hierarchical organization of the murine hematopoietic system.

We next assessed the repopulating capability and multi-lineage differentiation potential of *Trib2*^*−/−*^ HSPCs by transplantation of CD45.2^+^ whole-BM-nucleated cells (WT or *Trib2*^*−/−*^ donor) into lethally irradiated CD45.1^+^ mice (recipients). TRIB2 loss did not affect long-term engraftment of donor cells and their capability to fully reconstitute the blood system of recipients (Supplementary Figure S2A–C). Analysis of blood collected periodically from recipients showed no skewing of differentiation into myeloid and lymphoid lineages in the absence of TRIB2 during hematopoietic reconstitution (Supplementary Figure S2D). No significant difference was found in the HSPC populations in recipients transplanted with either genotype (Supplementary Figure S2E and F). We conclude that TRIB2 is dispensable for the maintenance of the hematopoietic system through differentiating HSPC populations that reside in the BM.

### TRIB2 regulates the proliferation of developing thymocytes

**Figure 2:**
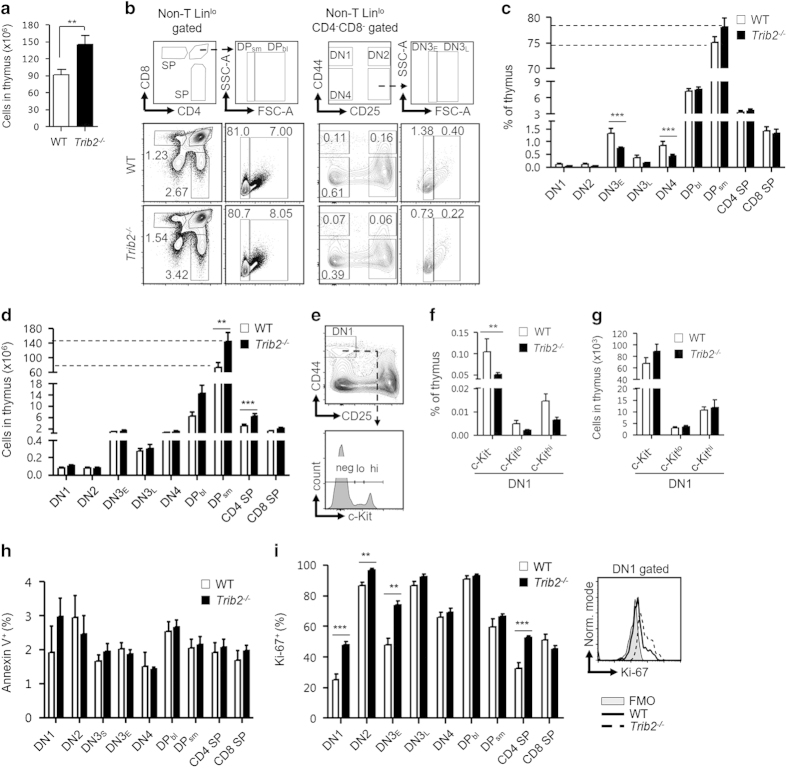
TRIB2 regulates the homeostasis of intrathymic T-cell development. (**a**) Thymic cellularity of WT (*n*=31) and *Trib2*^*−/−*^ (*n*=21) mice was counted by trypan blue exclusion after RBC lysis. (**b**) Flow cytometry of thymic subsets. The complete gating strategy is provided in Supplementary Figure S3. Each subset is indicated in the outlined areas (top row). The corresponded values in the representative staining profile of WT (middle row) and *Trib2*^*−/−*^ (bottom row) mice are frequency of thymus and graphed in **c**. CD4 SP, CD4^+^CD8^−^; CD8 SP, CD4^−^CD8^+^; DN1, Lin^lo^CD44^+^CD25^−^; DN2, Lin^lo^CD44^+^CD25^+^; DN3_E_, Lin^lo^CD44^−^CD25^+^FSC^lo^; DN3_L_, Lin^lo^CD44^−^CD25^+^FSC^hi^; DN4, Lin^lo^CD44^−^CD25^−^; DP_bl_, CD4^+^CD8^+^FSC^hi^; DP_sm_, CD4^+^CD8^+^FSC^lo^; FSC-A, forward scatter-area; SSC-A, side scatter-area. (**d**) Number of cells for each subset. (**e**) Further characterization of DN1 cells based on c-Kit surface expression. hi, high; lo, low; Neg, negative. DN1 subsets were graphed in frequency of thymus (**f**) and cell number (**g**). For **c**, **d**, **f** and **g**, *n*=7–8 per genotype. (**h**) Basal level of apoptosis of each thymic subset (*n*=3 per genotype) was determined by the surface expression of Annexin V after exclusion of DAPI-stained dead cells. (**i**) Intracellular level of Ki-67 across thymic subsets (*n*=4–5 per genotype) was measured by flow cytometry (left). An overlap of histogram (right) showed *Trib2*^*−/−*^ DN1 cells had higher level of Ki-67 compared with that of WT. FMO, Fluorescence Minus One; Norm, normalized. For statistical analyses, unpaired Student’s *t*-test was used for **a**, **i**, and two-way ANOVA was used for **c**, **d** and **f**. **P<0.01; ***P<0.001, all quantified data are presented as mean and s.e.m. ANOVA, analysis of variance; DAPI, 4′, 6-diamidino-2-phenylindole; RBC, red blood cell; SP, single positive.

T-cell development takes place in the thymus that is seeded constantly by T-cell progenitors originating in the BM. We found that *Trib2*^*−/−*^ mice had statistically significant higher thymic cellularity compared with WT mice (Figure 2a). This suggests dysregulation of thymopoiesis in the absence of TRIB2. We further examined thymic subsets along the αβ T-lineage developmental pathway by immunophenotyping. Non-T-lineage markers (CD11c, Gr-1, B220, Ter119 and NK1.1) were included in all the experimental analysis to exclude cells of other lineages present in thymus. Gating for lineage markers, including CD4 and CD8, were adjusted so as not to exclude c-Kit^+^ thymic progenitors that express low cell surface levels of lineage markers when defining immature CD4^−^CD8^−^ double-negative (DN) thymocytes [20] (Supplementary Figure S3). For gating of thymic subsets, DN3 thymocytes (Lin^lo^CD44^−^CD25^+^) were divided further into DN3_E_ (expected: FSC^lo^) and DN3_L_ (larger: FSC^hi^) subsets, based on cell size [21]. This is equivalent to characterization of DN3 into DN3a and DN3b subsets, based on CD27 marker, that corresponded to pre- and post-β selection [22]. Similarly, CD4^+^CD8^+^ double-positive (DP) thymocytes were divided further into DP_sm_ (small resting: FSC^lo^) and DP_bl_ (blasts: FSC^hi^) subsets [23]. As described [21, 24, 25], we found that DN3_E_ and DP_sm_ subsets were not proliferative as >99% were in G_0_/G_1_ phase and only 10–20% were CD71^+^. In contrast, DN3_L_ and DP_bl_ subsets were actively cycling as 60–70% were in S–G_2_/M phases and 90% were CD71^+^ (Supplementary Figure S4A–C). Our analysis of the thymic subsets showed that, in general, *Trib2*^*−/−*^ mice had a lower frequency of immature DN1–4 subsets, but the mature DP and single-positive subsets were unaffected compared with WT mice (Figure 2b and c). The higher cellularity of *Trib2*^*−/−*^ thymus was due to the significant increase of mature subsets (DP_sm_ and CD4 single positive) but not the immature DN1–4 subsets (Figure 2d). The DN1 (Lin^lo^CD44^+^CD25^−^) subset is heterogeneous and divided into DN1a–e subsets based on CD24 and c-Kit surface expression [26]. Here the DN1 subset was divided broadly into c-Kit^hi^ (DN1a/b), c-Kit^lo^ (DN1c) and c-Kit^−^ (DN1d/e) subsets (Figure 2e). *Trib2*^*−/−*^ c-Kit^−^ DN1 progenitors were present at a significantly lower frequency but had similar cell numbers compared with WT mice (Figure 2f and g). To rule out the possibility that lower frequency of *Trib2*^*−/−*^ DN1–4 subsets were due to increased apoptosis, we measured Annexin V expression and showed no difference between the two genotypes (Figure 2h). As *Trib2*^*−/−*^ mice had higher numbers of mature subsets that must be derived from immature subsets, we hypothesized that immature thymocytes were cycling faster and gave rise to more mature differentiated thymocytes in the absence of TRIB2. Ki-67 is a proliferation marker, expressed by cells in active cell cycle (G_1_–S–G_2_/M) but not by resting cells in G_0_ [27]. Indeed, intracellular staining of Ki-67 showed that *Trib2*^*−/−*^ DN1, DN2 and DN3_E_ subsets had significantly higher levels of Ki-67 compared with WT thymic subsets (Figure 2i), indicating that more *Trib2*^*−/−*^ developing thymocytes are in cycling state. This does not result in T-cell accumulation outside the thymus however (Supplementary Figure S5).

Cell cycle regulation is crucial for proper T-cell development. DN3 thymocytes must be briefly arrested (DN3_E_) at the G_0_/G_1_ cell cycle phases to allow V(D)J recombination to take place at the *Tcrb* locus, initiated by the RAG proteins [28, 29]. Functional *Tcrb* rearrangements lead to formation of pre-T-cell receptors where the signals drive the subsequent development of thymocytes to early DP stage. Given that *Trib2*^*−/−*^ developing thymocytes proliferate faster, we determined whether the loss of TRIB2 affects the cell cycle status of these subsets and *Tcrb* rearrangements. DNA staining showed that *Trib2*^*−/−*^ thymocytes at each stage of the T-cell development had a similar cell cycle profile compared with WT mice (Supplementary Figure S4A and B). This suggests that TRIB2 does not regulate the cell cycle phase progression, and that the increased cycling of *Trib2*^*−/−*^ developing thymocytes is likely due to changes in cell division kinetics at the steady state. Importantly, *Trib2*^*−/−*^ DN3_E_ thymocytes were arrested at G_0_/G_1_ phases such as WT DN3_E_ thymocytes. As such, analysis of *Tcrb* rearrangement involving the Jβ2.1 to Jβ2.7 gene segments demonstrated that *Trib2*^*−/−*^ thymocytes contain polyclonal *Tcrb* rearrangements similar to WT thymocytes (Supplementary Figure S4D).

### *Trib2*^*−/−*^ thymocytes are hypersensitive to 5-fluorouracil-induced cell death

**Figure 3:**
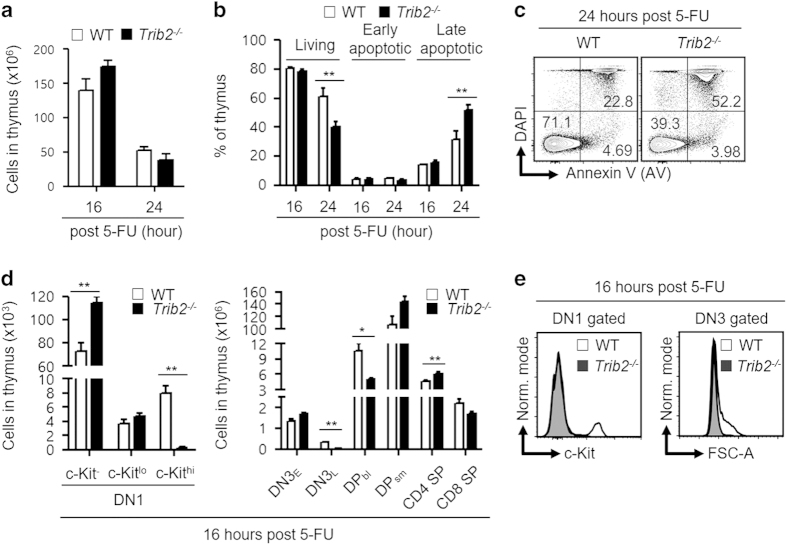
*Trib2*^*−/−*^ thymocytes are more susceptible to 5-FU-induced cell death. (**a**) Mice were treated with 5-FU (250 mg kg^−1^, i.p.) and sacrificed after 16 and 24 h to determine the thymic cellularity. (**b**) Apoptosis assays were performed to determine the fraction of living, early and late apoptotic cells in thymus at 16 and 24 h post treatment. (**c**) A representative staining profile (**b**) of 5-FU-treated WT and *Trib2*^*−/−*^ thymus. Living, DAPI^−^AV^−^; early apoptotic, DAPI^−^AV^+^; late apoptotic, DAPI^+^AV^+^. The values indicated in the outlined areas are frequency of thymus. (**d**) The cellularity of thymic subsets at 16 h post treatment. (**e**) Overlay of the histograms of DN1 and DN3 cells for c-Kit expression and FSC-A signals, respectively, showed loss of *Trib2*^*−/−*^ DN1c-Kit^+^ and DN3_L_ cells at 16 h post treatment. For **a**, **b** and **d**, *n*=5 per genotype per studied time point. For statistical analyses, unpaired Student’s *t*-test was used for **a** and two-way ANOVA was used for **b**, **d**. *P<0.05; **P<0.01, all quantified data are presented as mean and s.e.m. ANOVA, analysis of variance; DAPI, 4’, 6-diamidino-2-phenylindole; i.p., intraperitoneal.

5-fluorouracil (5-FU) is a standard chemotherapeutic drug widely used to treat cancers, as it targets rapidly cycling cells. Misincorporation of 5-FU metabolite into DNA during DNA replication initiates futile cycles of DNA excision, repair and further misincorporation that eventually lead to DNA strand breaks and cell death [30]. On account of that, we hypothesized that *Trib2*^*−/−*^ developing thymocytes which are highly proliferative to be more sensitive to 5-FU compared with WT thymocytes [31]. We performed a time-course study of *in vivo* 5-FU treatment. *Trib2*^*−/−*^ mice had significantly higher total thymic cellularity at steady state (Figure 2a); however, the significance difference lost at 16 and 24 h post treatment (Figure 3a). Significant increase of cell death (late apoptotic) was found in *Trib2*^*−/−*^ thymic cells at 24 h post treatment (Figure 3b and c). Notably, we demonstrated that DN3_L_ and DP_bl_ subsets, which are known to be proliferative (Supplementary Figure S4A–C), were significantly reduced in *Trib2*^*−/−*^ mice 16 h post treatment. DN3_E_ and DP_sm_ subsets, which were in resting state, were unaffected in treated WT and *Trib2*^*−/−*^ mice. Unexpectedly, *Trib2*^*−/−*^ c-Kit^hi^ DN1 thymic progenitors were also significantly reduced after 16 h of exposure to 5-FU, whereas *Trib2*^*−/−*^ c-Kit^−^ and c-Kit^lo^ DN1 progenitors were unaffected like WT mice (Figure 3d and e). c-Kit^hi^ DN1 progenitors were shown previously to exclusively exhibit a proliferative burst capacity by OP9-DL1 co-culture system compared with c-Kit^−^ and c-Kit^lo^ DN1 progenitors [26]. Interestingly, 5-FU was shown recently to preferentially induce apoptosis in c-Kit^hi^ HSCs that have rapid cell division kinetics compared with c-Kit^lo^ HSCs *in vitro* [32]. Overall, these data confirmed a role for TRIB2 in controlling the cell division kinetics of thymocytes and hence their enhanced sensitivity to 5-FU genotoxic drug.

### Acceleration of thymopoietic recovery in the absence of TRIB2 after genotoxic insult

**Figure 4:**
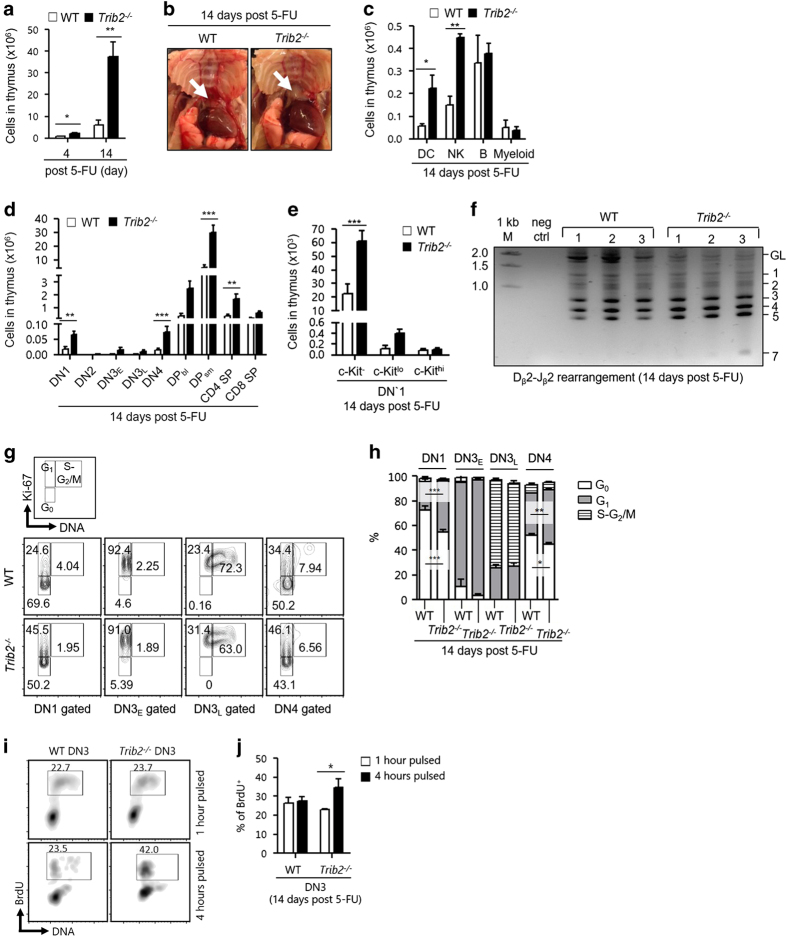
Thymopoietic recovery is accelerated in the absence of TRIB2 after genotoxic stress. (**a**) Thymic cellularity of mice after 4 and 14 days of 5-FU treatment. (**b**) The white arrow indicates representative thymus of the dissected WT and *Trib2*^*−/−*^mice. (**c**) The cellularity of non-T-lineage cells (DC, CD11c^+^; NK cells, Nk1.1^+^; B, CD19^+^B220^+^; Myeloid, Gr-1^+^CD11b^+^) in thymus (*n*=3 per genotype) were determined by flow cytometry. Cellularity for thymic subsets (**d**) and DN1 subsets (**e**). (**f**) PCR analysis (*n*=3 per genotype) of *Tcrb* rearrangement involving the D_β_2 to J_β_2 gene segments. GL denotes the position of the germline PCR product and number indicates the different rearrangements. 1 kb M, 1 kb DNA marker; neg ctrl, no template negative control. (**g**) Flow cytometry to determine the fraction of developing thymocytes in resting (G_0_) and active (G_1_–S/G_2_/M) cell cycle. Each phase is indicated in the outlined areas (top row). The corresponding values in the representative staining profile of WT (middle row) and *Trib2*^*−/−*^ (bottom row) mice (*n*=2–3 per genotype) are frequency of each phase and graphed in **h**. (**i**) The frequency of BrdU uptake by DN3 thymocytes after 1 and 4 h of pulsing. A representative staining profile of WT and *Trib2*^*−/−*^ mice (*n*=3 per genotype per studied time point) is shown here. The values indicated in the outlined areas are the frequencies of BrdU^+^ DN3 thymocytes and graphed in **j**. For **a**, **d** and **e**, *n*=9 per genotype per studied time point. 5-FU dosage for **a**–**e** was 250 mg kg^−1^, whereas for **f**–**j** it was 200 mg kg^−1^. For statistical analyses, unpaired Student’s *t*-test was used for **a** and two-way ANOVA was used for **c**–**e**, **h** and **j**. *P<0.05; **P<0.01; ^***^ *P*<0.001, all quantified data are presented as mean and s.e.m. ANOVA, analysis of variance.

Previous studies on the response of thymus to 5-FU-mediated genotoxic insult have shown that 5-FU induces apoptosis of thymocytes, thymic weight loss and damages to thymic architecture that is crucial for T-cell development [33, 34]. Thymic inner architecture exhibited morphological recovery on day 7 and was back to normal on day 10 post 5-FU injury in previous work. However, little is known about thymopoietic restoration. We compared thymopoietic recovery of WT and *Trib2*^*−/−*^ mice 4 and 14 days after a single dose of 5-FU administration. *Trib2*^*−/−*^ mice had higher thymic cellularity compared with WT mice at 4 and 14 days post treatment and the thymus was relatively bigger at 14 days post treatment (Figure 4a and b). Importantly, the higher cell count was not due to the increase of myeloid and B cells that do not normally develop in thymus (Figure 4c). Although WT and *Trib2*^*−/−*^ mice both had the hierarchy of thymic subsets restored to normal on day 14 compared with day 4 post 5-FU treatment (Supplementary Figure S6A–C), *Trib2*^*−/−*^ mice had significantly higher numbers of DN1 progenitors and the subsequent mature subsets (Figure 4d). This indicates that *Trib2*^*−/−*^ mice had accelerated thymopoietic recovery. At steady state, thymopoiesis is sustained mainly by c-Kit^hi^ DN1 progenitors [35]. However, these progenitors were absent in WT and *Trib2*^*−/−*^ thymus from day 1 to day 14 post 5-FU treatment (Supplementary Figure S6D). Instead, we found a significant increase of c-Kit^−^ DN1 progenitors in *Trib2*^*−/−*^ thymus suggesting expansion of these progenitors drives the accelerated recovery (Figure 4e).

We postulated that the acceleration of thymopoietic recovery in the absence of TRIB2 is due to the intrinsic highly proliferative nature of *Trib2*^*−/−*^ thymocytes. In developing thymocytes, proliferation and differentiation are tightly linked. Analysis of *Tcrb* rearrangements for the joining of Dβ2 to Jβ2 gene segments showed less immature thymocytes that have not undergone *Tcrb* rearrangements (germline (GL) band) present in *Trib2*^*−/−*^ thymus compared with WT thymus 14 days post 5-FU treatment (Figure 4f). This supports our finding that thymopoiesis was more active in treated *Trib2*^*−/−*^ mice. We further assessed the quiescent state of DN thymocytes through dual staining of DNA and Ki-67. Compared with WT DN thymocytes, significantly less *Trib2*^*−/−*^ thymocytes in DN1 and DN4 subsets were resting (G_0_) and correspondingly more *Trib2*^*−/−*^ thymocytes in these subsets were in cycling state (G_1_–S/G_2_–M; Figure 4g and h). This indicates that TRIB2 regulates the cell cycle entry of thymocytes. To expand beyond a static assessment of proliferation, we did time-course experiments of *in vivo* bromodeoxyuridine (BrdU) pulsing in WT and *Trib2*^*−/−*^ mice. DN3 thymocytes of both genotypes had similar uptake of BrdU after 1 h of pulsing; however, BrdU^+^ *Trib2*^*−/−*^ DN3 thymocytes were significantly increased after 4 h of pulsing (Figure 4i and j). This demonstrated that *Trib2*^*−/−*^ developing thymocytes had higher cell division kinetics and more DN3 thymocytes were available to uptake BrdU while replicating their DNA.

It is noteworthy that TRIB2 loss only affected thymopoietic recovery but not hematopoietic regeneration in BM. On day 14 post 5-FU treatment, blood cell counts of WT and *Trib2*^*−/−*^ mice were restored to normal with no measurable differences detected (Supplementary Figure S7A and B). However, more CD4^+^ and CD8^+^ T cells were present in the blood of *Trib2*^*−/−*^ mice though these were not statistically significant (Supplementary Figure S7C). Both genotypes had similar BM cellularity and HSPC populations, including common lymphoid progenitors that are lymphoid-restricted progenitors [36] (Supplementary Figure S7D–F). Hence, hematopoiesis in BM was restored normally in the absence of TRIB2.

### TRIB2 loss accelerates T-ALL via defective MAPK signaling

**Figure 5:**
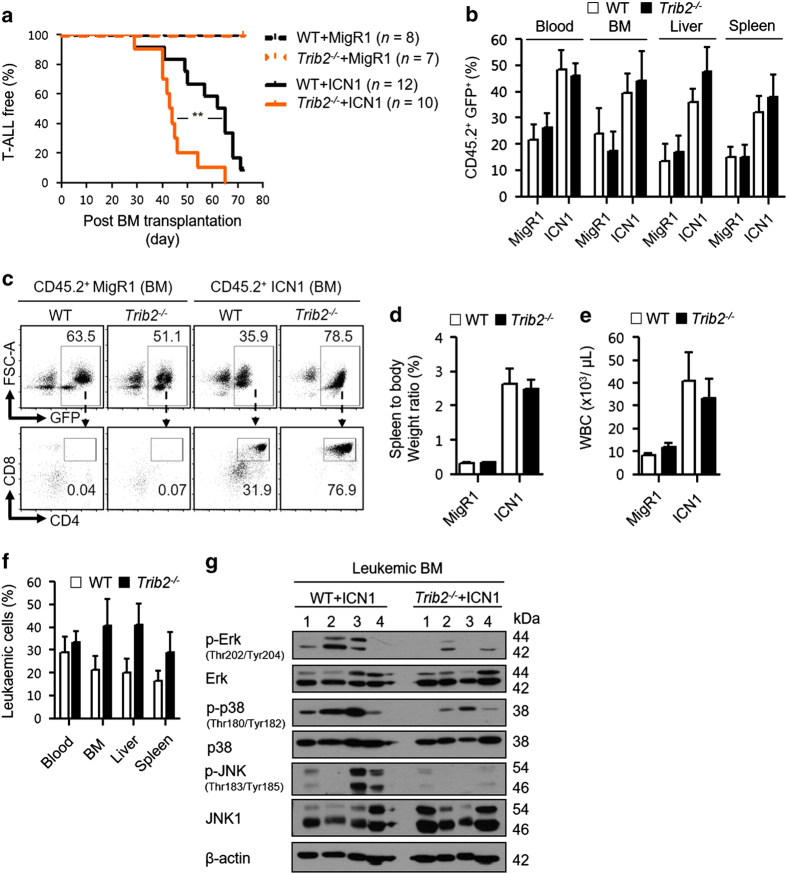
TRIB2 attenuates the leukemia initiation potential of NOTCH1. (**a**) Kaplan–Meier survival analysis of the lethally irradiated recipient (CD45.1^+^) mice transplanted with 5-FU-enriched WT or *Trib2*^*−/−*^ BM cells (donor: CD45.2^+^) transduced with either MigR1 control or ICN1 transgene. The number (*n*) of mice analyzed was from three independent experiments. Engraftment of transduced donor cells (CD45.2^+^GFP^+^) (**b**) and development of T-ALL leukemia (CD4^+^CD8^+^) (**c**) in moribund mice were verified by flow cytometry analysis. The values in the outlined areas (**c**: bottom row) are frequency of leukemic cells in the BM of moribund mice. Leukemic burden was assessed by the spleen to body weight ratio (**d**) and WBC counts (**e**) of moribund mice. (**f**) Leukemic infiltration was assessed by measurement of the frequency of leukemic cells in various organs of moribund mice. (**g**) Activation of MAPK signaling in T-ALL was determined by western blotting for p-Erk, total Erk, p-p38, total p38, p-JNK, JNK1 and β-actin signals in the leukemic BM (*n*=4 per group). Signals shown were developed from triplicate immunoblots. For **a**, Log-rank test was used to compare the survival curves. **P=0.0053, all quantified data are presented as mean and s.e.m. GFP, green fluorescent protein; WBC, white blood cell.

As TRIB2 appears to negatively regulate the proliferation of developing thymocytes, the normal counterpart of T-ALL, we examined the role of TRIB2 in T-cell leukemogenesis using a NOTCH1-induced T-ALL BM transplantation mouse model [37]. WT and *Trib2*^*−/−*^ BM cells were transduced with an empty vector control (MigR1) or a retroviral vector encoding an intracellular *Notch1* transgene (ICN1). The cells, which were CD45.2^+^, were then transplanted into lethally irradiated CD45.1^+^ recipient mice to monitor the development of T-ALL. All experimental groups were verified by *Trib2* genotyping analysis of the transplanted moribund mice (Supplementary Figure S8). Mice transplanted with ICN1-transduced *Trib2*^*−/−*^ donor cells succumbed to T-ALL disease with a shorter latency (median survival of 43.5 days), whereas mice transplanted with ICN1-transduced WT donor cells had a median survival of 63.5 days. Kaplan–Meier survival analysis for these two groups was significantly different (Figure 5a), indicating that T-ALL onset driven by NOTCH1 overexpression was accelerated in the absence of TRIB2. Green fluorescent protein expression was used as a marker for transduced donor cells and analysis showed similar engraftment levels of transduced WT and *Trib2*^*−/−*^ donor cells in the recipients from all groups across different analyzed organs (Figure 5b). TRIB2 loss did not alter the phenotype of murine NOTCH1-induced T-ALL (Figure 5c). Despite a difference of 20 days for disease onset, mice that succumbed earlier to *Trib2*^*−/−*^ T-ALL had a similar degree of leukemic burden (Figure 5d and e) but exhibited a trend of higher organ infiltration of leukemic cells (Figure 5f) compared with mice that succumbed to WT T-ALL. We have ruled out that the T-ALL phenotype is not due to an increase in *Trib1* expression, as *Trib1* expression levels do not change upon knockdown of *Trib2* in T-ALL or AML cell lines (Supplementary Figure S9; Supplementary Table S2). Tribbles family (TRIB1–3) is known to be associated with MAPK signaling and required for the activation of ERK, JNK and p38 [38–41]. Western blotting for MAPK signals in leukemic infiltrated BM samples showed impaired activation of ERK, JNK and p38 in *Trib2*^*−/−*^ T-ALL compared with WT T-ALL (Figure 5g).

### *Trib2* expression levels distinguish molecular subtypes of T-ALL and correlate with MAPK signaling

**Figure 6:**
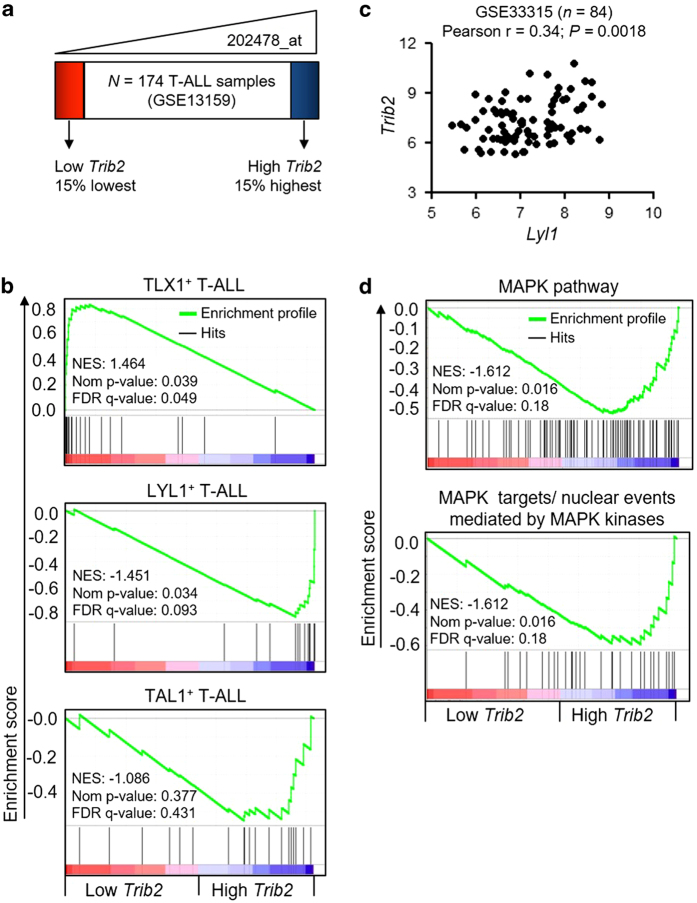
*Trib2* levels distinguish molecular subtypes of human T-ALL and associate with MAPK signaling. (**a**) GSEA was performed to compare human T-ALL samples from GSE13159 data set [42] that expressed low and high *Trib2* (*n*=26 per group) for enrichment of human T-ALL molecular subtypes (**b**) and canonical pathways (**d**). NES, normalized enrichment score; Nom, nominal. (**c**) Correlation between *Trib2* (average of 202478_at and 202479_s_at) and *Lyl1* (210044_s_at) expressions were examined in 84 human T-ALL samples from GSE33315 data set [44]. For **c**, Pearson correlation test was used to examination association between *Trib2* and *Lyl1* expressions.

As TRIB2 loss accelerated T-ALL onset in our experimental model, we performed gene set enrichment analysis (GSEA) to compare gene expression profiles of low and high *Trib2* expression from a database (GSE13159) derived from 174 T-ALL patient samples (Figure 6a) [42]. GSEA analysis showed these two groups, defined by *Trib2* expression, were of distinct molecular subtypes of T-ALL (Figure 6b). Low *Trib2*-expressing T-ALL group was enriched with gene set associated with TLX1^+^ T-ALL (cortical mature T-ALL), whereas high *Trib2*-expressing T-ALL group had upregulation of LYL1^+^ T-ALL (early immature T-ALL) gene set [43]. TAL1^+^ T-ALL gene set was not enriched in either group. A significant positive correlation between *Trib2* and *Lyl1* expressions was confirmed in an independent T-ALL data set (GSE33315) [44] (Figure 6c). In our experimental model, *Trib2*^*−/−*^ T-ALL leukemic cells exhibited deficiencies in MAPK signaling. In accordance with this, using GSEA analysis of low and high *Trib2* expressed human T-ALL groups, MAPK signaling was found to be upregulated in the high *Trib2*-expressing T-ALL group (Figure 6d). Hence, impaired activation of MAPK signaling in the absence of TRIB2 contributed to the increased aggressiveness of NOTCH1-induced murine T-ALL disease that recapitulated the immunophenotypes of human cortical mature T-ALL. Our analysis suggests that TRIB2 possesses tumor-suppressive functions important for T-ALL.

## Discussion

Our data provide strong evidence that TRIB2 is a novel regulator of thymopoietic proliferation, important in the response to stress and the pathogenesis of T-ALL. We have established a previously unrecognized role for TRIB2 as a novel regulator for cellular proliferation of developing thymocytes. At steady state, *Trib2*^*−/−*^ developing thymocytes proliferated faster and gave rise to more mature thymocytes. The accelerated proliferation of *Trib2*^*−/−*^ developing thymocytes conferred hypersensitivity to 5-FU-induced cell death. Following genotoxic insult, *Trib2*^*−/−*^ mice exhibited accelerated thymopoietic recovery due to expansion of c-Kit^−^ DN1 progenitors and high cell division kinetics of developing thymocytes. In the absence of TRIB2, NOTCH1 is more potent in driving T-ALL initiation and the increased aggressiveness of *Trib2*^*−/−*^ T-ALL was enhanced by impaired activation of MAPK signaling. Human T-ALL can be distinguished based on *Trib2* expression levels and, in accordance with our experimental data, correlate with MAPK signaling in high *Trib2-*expressing T-ALLs.

Here we show TRIB2 as the first member of the mammalian Tribbles family to have a proliferative role in the context of developing thymocytes during T-cell development. TRIB1 was reported previously to negatively regulate proliferation of human aortic smooth muscle cells *in vitro* via interaction with MKK4 [45]. TRIB2 regulates cell cycle entry/exit and hence the cell division kinetics of developing thymocytes. We found that the dysregulated proliferation of *Trib2*^*−/−*^ developing thymocytes does not affect *Tcrb* rearrangement, a key event in early T-cell differentiation. Furthermore, loss of TRIB2 did not affect terminal maturation of developing thymocytes. Intriguingly, the increased proliferation of developing thymocytes, in the absence of TRIB2, does not affect the percentage of peripheral T cells. Previous literature has shown that mice overexpressing CD69 have increased thymic SP subsets but a reduction in the number of T cells in the peripheral lymphoid organs [46]. Our data suggest that TRIB2 may impact on thymic selection (DPsm to CD69^+^ DP transition) or thymic T-cell export. It is also likely that TRIB2 has a distinct role in mature T-cell biology and function because *Trib2* expression distinguishes CD4^+^ from CD8^+^ peripheral T-cells [23]. This could be further explored using appropriate infection and immunization models.

Thymopoietic recovery is critical to replenish the T-cell repertoire in order to reconstitute cellular immunity due to T-cell depletion in the clinical setting. At steady state, human T-cell repertoire is established during early childhood as removal of the thymus in children after 6 months of age does not cause overt immunodeficiency [47]. However, many medical conditions such as chemo- and irradiation therapy, infection and graft versus host disease can cause T-cell depletion, thymus insult and thus impaired T-cell immunity in patients [48]. Our study showed that TRIB2 limits the thymopoietic recovery after 5-FU injury. The reason underlying this is unclear but tight regulation of thymopoietic restoration has been proposed to prevent adverse consequences such as leukemic transformation of T-cells or autoimmunity [48].

We showed that TRIB2 functions to suppress T-cell leukemogenesis induced by NOTCH1 overexpression. A role for TRIB2 in T-ALL maintenance was demonstrated by Sanda et al [13] , as knockdown of *Trib2* in a panel of TAL1-positive human T-ALL cell lines induced apoptosis and inhibited cell growth. This appears to be consistent with the role of TRIB2 in other solid [49–51] and hematological [9] malignancies that overexpression of TRIB2 confers growth and survival advantages to tumor cells. However, we provide strong evidence that TRIB2 has opposing roles in the initiation and potency of T-ALL. In the absence of TRIB2, the latency of NOTCH1-induced murine T-ALL was shortened significantly. We previously reported elevated *Trib2* levels in a cohort of pediatric T-ALLs that contain NOTCH1/FBXW7 mutations compared with WT T-ALLs [11]. However, T-ALL is a heterogeneous disease and can be classified into different molecular subtypes, although aberrant NOTCH1 signaling is the unifying feature in all the subtypes. Hence, we performed GSEA analysis in this study to show *Trib2* is high in early immature (LYL1^+^) T-ALLs that are arrested at the DN stage of thymocyte development and show a transcriptional program related to HSCs and myeloid progenitors [44, 52]. In turn, *Trib2* is low in TLX1^+^ human T-ALLs that have a mature cortical DP phenotype where the transformed cells have committed to the T-cell lineage [43]. We propose that the role of TRIB2 in different subtypes of human T-ALL depends on the stage at which thymic progenitors undergo malignant transformation, and if they have committed to T-cell lineage.

There is a strong correlation with *Trib2* and MAPK signaling in human T-ALL as shown by GSEA analysis. Previously, the activation of ERK and p38 was shown to inversely correlate with aggressiveness of T-ALL in a model of T-ALL cell dormancy [53]. Furthermore, drug treatment of established human T-ALL cell lines was shown to induce cell death via activation of p38 and JNK [54–56]. Thus, activation of MAPK signaling appears to be a limiting factor, at least, in the context of T-ALL. TRIB family members act as scaffold proteins for the binding of MAPK signaling complexes and hence modulate their activity. All TRIB family members have the conserved MEK1-binding motif and have been shown to interact with MEK1 [38]. In human T-ALLs, upregulation of ERK1/2 signaling was found in 38% of T-ALL patients [57] and MEK/ERK can be activated due to RAS mutations, found in ~10% of T-ALL patients [58]. Targeting of MEK/ERK signaling by MEK inhibition was shown to be effective in treating KRAS-mutated T-ALL in a murine model [59]. The upregulation of MAPK signaling in specific subtypes of T-ALL has not been studied and remains unclear. Here we show that *Trib2* expression is high in LYL1^+^ immature T-ALLs enriched with MAPK signaling, and our experimental data show defective MAPK signaling in the absence of TRIB2. Thus, it appears that TRIB2 functions to control T-ALL via MAPK modulation.

We and others [7, 60] have shown that *Trib2* is a myeloid oncogene when overexpressed in a murine BM transplant model. Here loss of TRIB2 potentiated murine T-ALL induced by a T-cell oncogene. Given that *Trib2* is expressed highest in normal T cells [19], it is not surprising that overexpression of *Trib2* does not drive T-ALL in the BM transplant model, and our data would suggest that TRIB2 functions to suppress T-ALL. Our data provide insight into the understanding of the opposing leukemogenic roles of TRIB2 in myeloid and lymphoid leukemia. However, the mechanisms that underlie whether TRIB2 acts as an oncogene or tumor suppressor are not well understood. It may be linked to its function in cell proliferation and MAPK pathway modulation, which could be cell context specific. Anti- and pro-proliferative effects connected with MAPK signaling and TRIB1 have been previously demonstrated in different cell contexts [38, 45].

To conclude, our studies have shown that TRIB2 is a novel regulator for the cellular proliferation of developing thymocytes, and the response to genotoxic and oncogenic stress. Although the loss of TRIB2 resulted in hypersensitivity to genotoxic stress, thymocytes recovered faster due to the inherent increased proliferation in the absence of TRIB2. These characteristics however are pro-oncogenic, as accelerated T-ALL was observed in the absence of TRIB2. Our findings provide a novel link between proliferative stress and TRIB2 in developing thymocytes, and may prove relevant for clinical practice in hematology and oncology.

## Materials and Methods

### Antibodies

The following antibodies, from BioLegend (San Diego, CA, USA) and eBioscience (San Diego, CA, USA), were used to stain cells for flow cytometry analysis: anti-B220 (RA3–6B2), anti-BrdU (BU20A), anti-CD3 (17A2), anti-CD4 (RM4–5 and GK1.5), anti-CD8α (53-6.7), anti-CD11b (M1/70), anti-CD11c (N418), anti-CD16/CD32 (93), anti-CD19 (eBio1D3), anti-CD25 (PC61.5), anti-CD34 (RAM34), anti-CD44 (IM7), anti-CD45.2 (104), anti-CD48 (HM48-1), anti-CD71 (R17217) anti-CD150 (TC15-12F12.2), anti-c-Kit (2B8), anti-Gr-1 (RB6–8C5), anti-IL-7Rα (A7R34), anti-Ki-67 (SolA15), anti-NK1.1 (PK136), anti-Sca-1 (D7) and anti-Ter119 (TER-119).

The following antibodies were used for western blotting: anti-p38 (D13E1), anti-phospho-p38 (D3F9), anti-p44/42 (137F5), anti-phospho-p44/42 (D13.14.4E), anti-JNK1 (2C6), anti-phopho-JNK and anti-β-actin (AC-15). Antibodies used to immunoblot MAPK signaling were from Cell Signaling Technology (Danvers, MA, USA) and anti-β-actin antibody was from Sigma-Aldrich (St Louis, MO, USA).

### Mice

*Trib2*^*−/−*^ (B6; 129S5-*Trib2*^*tm1Lex*^) mice, backcrossed onto C57BL/6, were bred and housed in the biological service unit of the University of Glasgow. Mice (B6.SJL-*Ptprc*^*a*^ *Pepc*^*b*^/ BoyJ) used in BM transplantation as recipients were bred and housed in the Beatson Biological Service and Research Units. Age-matched (6–14 weeks old) mice of both genders were used for comparison between WT and *Trib2*^*−/−*^ genotypes.

### Blood cell counts

Mice were sacrificed by carbon dioxide inhalation and blood was subsequently collected by cardiac puncture. Collected blood was analyzed by Hemavet 950 (Drew Scientific, Miami Lakes, FL, USA) to generate complete blood counts and white blood cell differential counts.

### Cell preparation, surface staining and flow cytometry

Pelvises, femurs and tibias collected from euthanized mice were dissected free of muscle tissues and tendons. The bones were crushed in 2% fetal bovine serum supplemented phosphate-buffered saline (PBS) solution using a mortar and pestle. The resulting cell suspension was filtered through a cell strainer. Thymus was isolated and grinded directly onto a cell strainer to generate single-cell suspensions. Red blood cell in blood, BM and thymus cell suspensions were lysed by homemade Ammonium-Chloride-Potassium Lysing Buffer containing 10 mm KHCO_3_, 150 mm NH_4_Cl and 0.1 mm EDTA. Live-cell count for BM and thymus was then performed by trypan blue exclusion.

Lineage antibody cocktail for BM cell surface staining included anti-CD3, anti-CD4, anti-CD8, anti-B220, anti-CD11b, anti-Gr-1 and anti-Ter119 antibodies. For thymus, non-T-lineage antibody cocktail included anti-CD11c, anti-B220, anti-Gr-1, anti-NK1.1 and anti-Ter119 antibodies. Antibodies used for flow cytometry were conjugated either with single fluorochrome or with biotin and revealed by fluorochrome-labeled streptavidin. All the cell populations identified by flow cytometry were defined in the related figure legends.

Unless indicated otherwise, antibody staining and washing of single-cell suspensions were done in 2% fetal bovine serum supplemented PBS solution. UltraComp eBeads (eBioscience) was used to prepare single-color compensation controls in multicolor flow cytometry experiments. Flow cytometry was performed using BD FACSCanto II system, equipped with blue, red and violet lasers (BD Biosciences, San Jose, CA, USA). Aggregates were excluded by forward scatter (FSC) height versus area signals. The singlet population was then gated by FSC area versus side scatter-area signals to exclude dead cells and debris. Whenever possible, dead cells were also excluded by 4′, 6-diamidino-2-phenylindole or propidium iodide staining. FlowJo (Tree Star, Ashland, OR, USA), was used to analyze flow cytometry data. Fluorescence Minus One controls were used to facilitate gating as appropriate.

### *In vivo* 5-FU treatment

On the drug administration day, mice were weighed to calculate the weight-adjusted dosage, as indicated in the related figure legends, and FU 50 mg ml^−1^ Solution (Accord Healthcare Limited, North Harrow, Middlesex, UK) was pre-diluted with PBS solution and was administered by intraperitoneal injection.

### Apoptosis assay

Thymus was collected from euthanized mice after the indicated period of 5-FU treatment and single-cell suspension was prepared as described above. Cells were stained with Annexin V-fluorescein isothiocyanate and 4’, 6-diamidino-2-phenylindole in Hank’s balanced salt solution, and analyzed by flow cytometry.

### Detection of endogenous *Tcrb* rearrangements

Total thymocytes were digested overnight with proteinase K in published lysis buffer [61] at 56 °C. Samples were then heated at 95 °C for 30 min to inactivate proteinase K. DNA was purified further by phenol extraction and ethanol precipitation. V(D)J rearrangements of the *Tcrb* locus were amplified by FastStart High Fidelity PCR system (Roche, Indianapolis, IN, USA), with 25 ng of DNA input, using published primers [62]. Primer sequences and the annealing temperatures for each amplification were listed in Supplementary Table S3. PCR products were separated on 1.5% agarose gel, detected by SafeView Nucleic Acid Stain (NBS Biologicals, Huntingdon, Cambridgeshire, UK), and imaged using ChemiDoc XRS system (Bio-Rad, Hercules, CA, USA).

### Intracellular staining of Ki-67 and cell cycle analysis

Thymocytes were stained with surface markers to allow subset identification, and then fixed in 4% weight/volume paraformaldehyde-PBS solution for 20 min at room temperature, permeabilized in 0.1% (weight/volume) saponin-PBS solution for 15 min at room temperature, followed by staining with anti-Ki-67 antibody in the permeabilization solution and analyzed by flow cytometry.

To distinguish resting (G_0_) from active (G_1_–S/G_2_/M) cell cycle, surface stained thymocytes were fixed and permeabilized by using BD Cytofix/Cytoperm Kit (BD Biosciences), followed by staining with anti-Ki-67 antibody and DNA selective Vybrant DyeCycle Violet Stain (Life Technologies, Eugene, OR, USA) and analyzed by flow cytometry.

### *In vivo* BrdU pulsing and detection of incorporated BrdU

On day 14 post 5-FU treatment, mice were pulsed with 1 mg BrdU-labeling reagent (Invitrogen, Camarillo, CA, USA), by intraperitoneal injection, for 1 or 4 h. Thymus was collected from euthanized mice and single-cell suspension was prepared. Surface-stained thymocytes were fixed in BD Cytofix/Cytoperm Buffer for 30 min on ice and stored at −80 °C for overnight in 10% dimethylsulfoxide supplemented fetal bovine serum to permeabilize the nuclear membrane. Cells were then thawed at 37 °C, fixed with BD Cytofix/Cytoperm Buffer for an additional 10 min on ice and treated with DNase I (30 μl per 1×10^6^ cells) from STEMCELL Technologies (Vancouver, BC, Canada) for 1 h at 37 °C. Cells were stained with anti-BrdU antibody and Vybrant DyeCycle Violet Stain (Life Technologies) in BD Perm/Wash Buffer (San Jose, CA, USA) and analyzed by flow cytometry.

### Bone marrow transduction and transplantation

Retroviral transduction of WT and *Trib2*^*−/−*^ BM cells, and subsequent transfer of these cells into lethally irradiated recipients (B6.SJL) were performed as described previously [7]. In brief, BM cells were collected from 6–8-week-old mice 4 days after administration of 5-FU (250 mg kg^–1^, intraperitoneally) and retrovirally transduced *ex vivo*, with MigR1 or MigR1-ICN1, in the presence of interleukin (IL)-3 (10 ng ml^−1^), IL-6 (10 ng ml^−1^) and stem cell factor (100 ng ml^−1^). Retroviral supernatants for MigR1 and MigR1-ICN1 were generated by transient transfection of 293T cells (together with pCMV-Gag-Pol packaging vector and pCMV-VSV-G envelope vector) and titered with 3T3 cell as previously described [10]. MigR1 and MigR1-ICN1 retroviral supernatants with equal titers were used to ensure similar transduction efficiency across three independent transplant experiments. Cells (0.25–0.5×10^6^) were then injected intravenously into lethally irradiated (2×4.25 grays fractionated doses were given 3 h apart) recipients. Mice were monitored by periodic tail vein bleedings 3 weeks after transplantation. Mice were sacrificed when they showed any of the following symptoms of disease: severe cachexia, lethargy, white blood cell count >20×10^6^ cells per ml and hunching. Healthy controls were sacrificed at the end of experiment when all mice in the test groups succumbed to disease.

### GSEA analysis

Gene expression profiles of T-ALL and healthy BM samples from GSE13159 data set [42] were downloaded from the Leukemia Gene Atlas platform [63]. On the basis of 202478_at feature, *Trib2* expression of low *Trib2* T-ALL group ranged from 3.602 to 5.370, whereas high *Trib2* T-ALL group ranged from 8.811 to 11.103. The mean value of *Trib2* for healthy BM samples (*n*=73) was 6.172. GSEA [64] was performed using the default settings. Gene sets from the Molecular Signatures Database v5.0 of GSEA (Broad Institute of MIT and Harvard, Cambridge, MA, USA) were used. These included the 1330 gene sets in C2: CP collection for canonical pathways and the three gene sets in C2: CGP collection (M4175, M2059 and M1007) for T-ALL molecular subtypes.

### Western blotting

Total BM cells collected from euthanized mice were lysed in homemade Radio-Immunoprecipitation Assay buffer (50 mm Tris buffer (pH7.4), 150 mm NaCl, 0.5% igepal CA 630, 0.25% sodium deoxycholate and 1 mm EDTA). Protein lysates were resolved by sodium dodecylsulfate polyacrylamide gel electrophoresis using 12% gels and transferred to 0.45-μm nitrocellulose membranes. The membranes were probed with primary antibodies followed by horseradish peroxidase-conjugated secondary antibodies. Stripping buffer (Thermo Scientific, Rockford, IL, USA) was used as appropriate for secondary probing.

### Statistics

GraphPad Prism (version 5.03; La Jolla, CA, USA) was used for statistical analysis and graphing. An unpaired, two-tailed, Student’s *t*-test was applied for comparison of two groups. For analysis of multiple groups between WT and *Trib2*^*−/−*^ genotypes, two-way analysis of variance with Bonferroni post tests was applied. Log-rank test was applied for survival curve comparison. Pearson correlation test was applied to examine association between *Trib2* and *Lyl1* expressions of T-ALL samples from GSE33315 data set [44]. This data set was downloaded from Gene Expression Omnibus and Robust Multichip Average method was used for data normalization. D’Agostino-Pearson omnibus test confirmed the T-ALL samples had normal distributions of *Trib2* and *Lyl1* values. Statistical significance of differences was attained when *P*-value <0.05 and was indicated in the related graphs.

### Study approval

All mouse experiments were approved by UK Animal Ethical Committees and performed according to UK Home Office project license (Animal (Scientific Procedures) Act 1986) guidelines.

## Notes

Supplementary Table S3

Supplementary Method
