# Supplementary material for: TRIB2 regulates normal and stress-induced thymocyte proliferation
Source: Cell Discov. 2016 Mar 15;2:15050–. doi: 10.1038/celldisc.2015.50 (PMC4860960; doi:10.1038/celldisc.2015.50)
Supplement: Supplementary Figure S1 [file celldisc201550-s1.pdf]

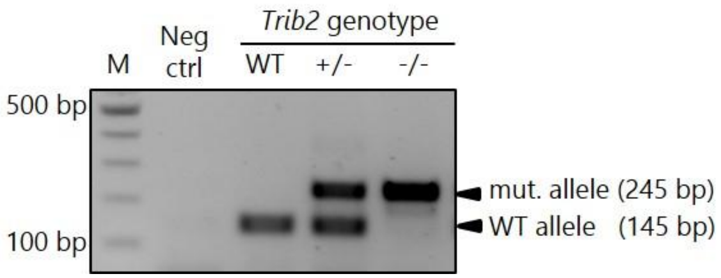

Figure S1. *Trib2* genotyping by genomic DNA PCR. A representative gel electrophoresis image showing resolution of PCR products amplified from WT, *Trib2*$^{-/+}$ and *Trib2*$^{-/-}$ mice using primers listed in Table S1. M, marker; Neg ctrl, no template negative control; mut, mutant.
