# Supplementary material for: TRIB2 regulates normal and stress-induced thymocyte proliferation
Source: Cell Discov. 2016 Mar 15;2:15050–. doi: 10.1038/celldisc.2015.50 (PMC4860960; doi:10.1038/celldisc.2015.50)
Supplement: Supplementary Figure S2 [file celldisc201550-s2.pdf]

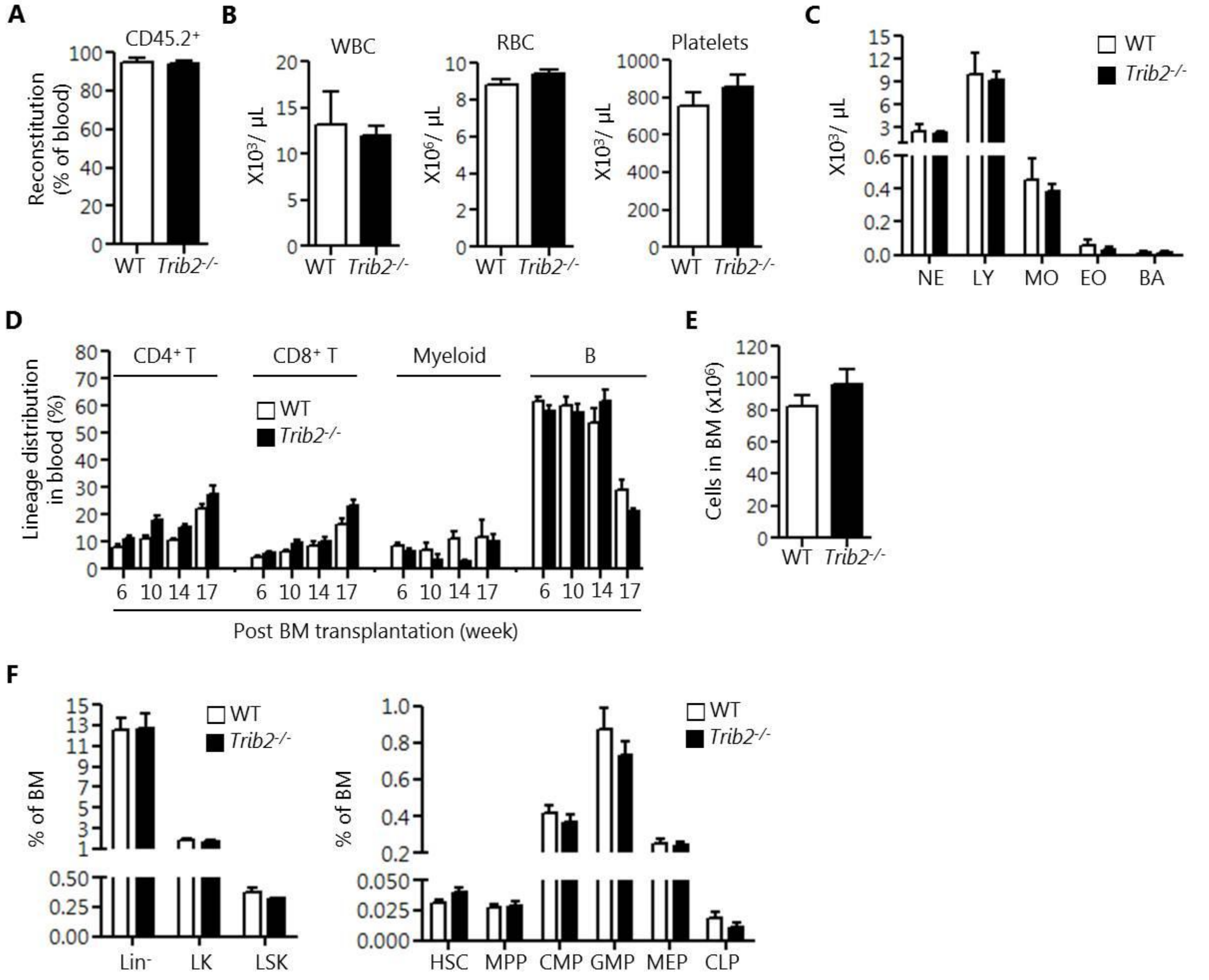

Figure S2. TRIB2 loss does not affect the repopulation capability of BM HSPCs. Lethally irradiated CD45.1$^+$ mice were transplanted with CD45.2$^+$ WT (n = 5) or *Trib2*$^{-/-}$ (n = 5) whole BM cells, and sacrificed after 17 weeks of transplantation. **(A)** Engraftment of donor cells was determined by measurement of CD45.2 expression in the blood of transplanted mice. Complete blood counts **(B)** and WBC differential counts **(C)** of these two groups of mice were determined by hematology analyzer. NE, neutrophils; LY, lymphocytes; MO, monocytes; EO, eosinophils; BA, basophils. **(D)** The distribution of donor derived mature myeloid, B and T cells in the blood of these mice was measured by flow cytometry. **(E)** BM cellularity was counted by trypan blue exclusion after RBC lysis of the cell suspension **(F)** Immunophenotyping and quantification of donor derived HSPCs (HSC, MPP, CMP, GMP, MEP and CLP) in BM. Lin, lineage; LK, Lin$^-$c-

Kit^+ cells; LSK, $Lin^-Sca\text{-}1^+c\text{-}Kit^+$ cells; HSC: LSK $CD150^+CD48^-$; MPP: LSK $CD150^-CD48^-$; CMP: LK $CD34^+CD16/32^{lo}$; GMP: LK $CD34^+CD16/32^{hi}$; MEP: LK $CD34^-CD16/32^-$; CLP: $Lin^-IL\text{-}7R\alpha^+$. All quantified data are presented as mean and SEM.
