# Supplementary material for: TRIB2 regulates normal and stress-induced thymocyte proliferation
Source: Cell Discov. 2016 Mar 15;2:15050–. doi: 10.1038/celldisc.2015.50 (PMC4860960; doi:10.1038/celldisc.2015.50)
Supplement: Supplementary Figure S3 [file celldisc201550-s3.pdf]

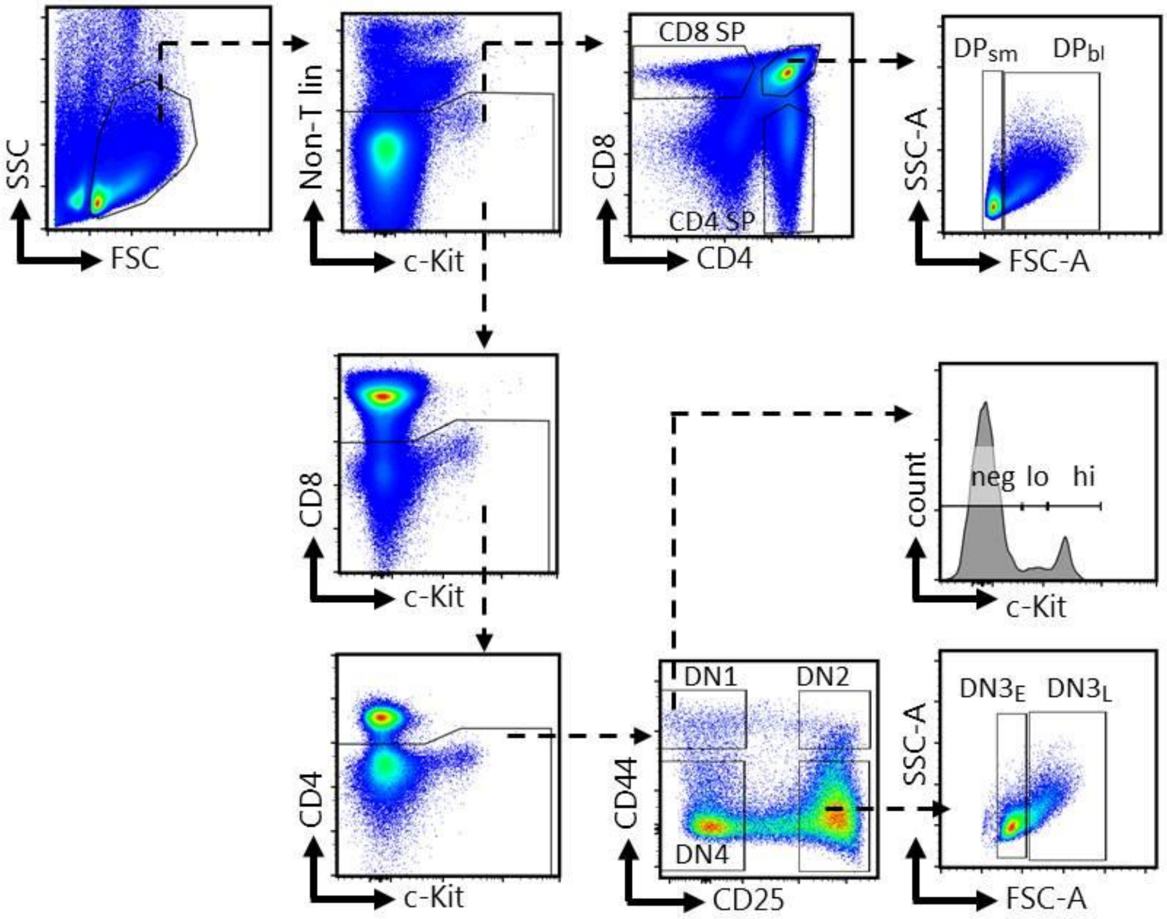

Figure S3. Gating strategy to identify thymic subsets. FSC-A, forward scatter-area; SSC-A, side scatter-area; DN1: $Lin^{lo}CD44^{+}CD25^{-}$; DN2: $Lin^{lo}CD44^{+}CD25^{+}$; $DN3_E$: $Lin^{lo}CD44^{-}CD25^{+}FSC^{lo}$; $DN3_L$: $Lin^{lo}CD44^{-}CD25^{+}FSC^{hi}$; DN4: $Lin^{lo}CD44^{-}CD25^{-}$; DP_{bl}: $CD4^{+}CD8^{+}FSC^{hi}$; DP_{sm}: $CD4^{+}CD8^{+}FSC^{lo}$; CD4 SP: $CD4^{+}CD8^{-}$; CD8 SP: $CD4^{-}CD8^{+}$.
