# Supplementary material for: TRIB2 regulates normal and stress-induced thymocyte proliferation
Source: Cell Discov. 2016 Mar 15;2:15050–. doi: 10.1038/celldisc.2015.50 (PMC4860960; doi:10.1038/celldisc.2015.50)
Supplement: Supplementary Figure S4 [file celldisc201550-s4.pdf]

A

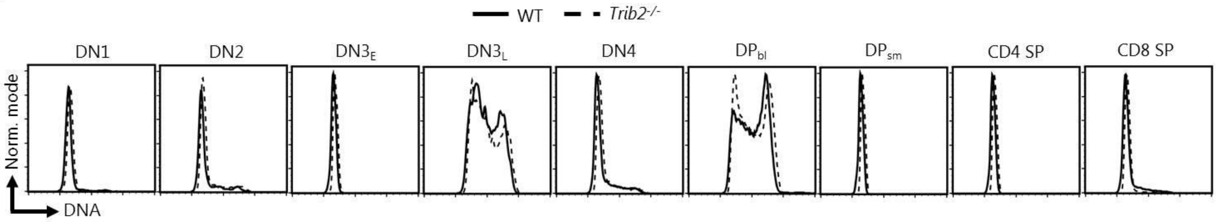
WT
Trib2-/-
DN1
DN2
DN3E
DN3L
DN4
DPbl
DPsm
CD4 SP
CD8 SP
Norm. mode
DNA

B

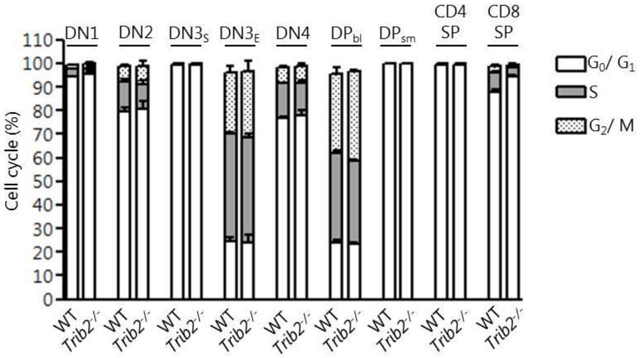
DN1
DN2
DN3S
DN3E
DN4
DPbl
DPsm
CD4 SP
CD8 SP
Cell cycle (%)
G0/ G1
S
G2/ M
WT
Trib2-/-

C

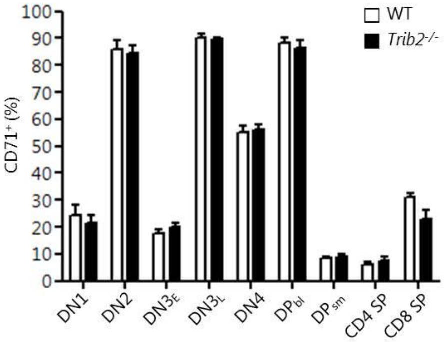
WT
Trib2-/-
CD71+ (%)
DN1
DN2
DN3E
DN3L
DN4
DPbl
DPsm
CD4 SP
CD8 SP

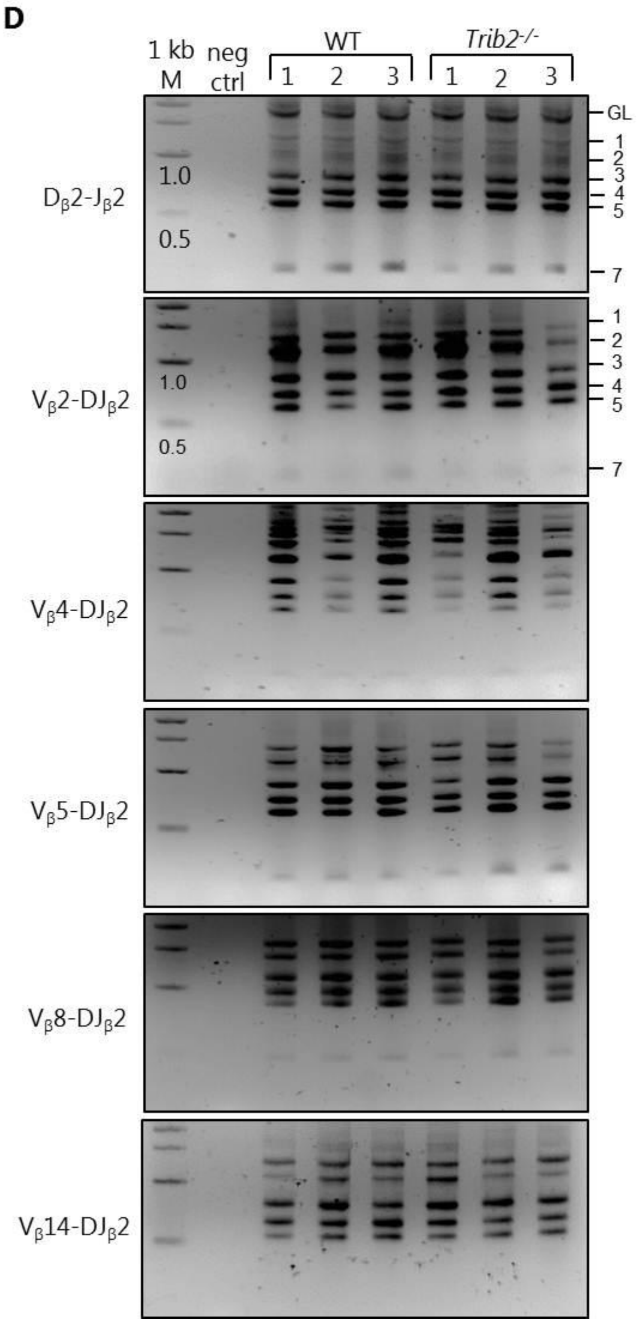

Figure S4. Characterization of WT and *Trib2*$^{-/-}$ thymic subsets. Overlay of a representative cell cycle profile **(A)** of WT and *Trib2*$^{-/-}$ thymic subsets and the fractions of cell cycle phases were graphed in **(B)**. Norm, normalized. **(C)** Quantification of CD71 expression of each thymic subset. **(D)** Endogenous *Tcrb* rearrangements of WT and *Trib2*$^{-/-}$ total thymocytes were analyzed by genomic DNA PCR. GL denotes the position of the germline PCR product and number indicates

the different rearrangements involving the $J_{\beta}2.1$ to $J_{\beta}2.7$ gene segments. 1 kb M, 1 kb DNA marker; neg ctrl, no template negative control. For **A-D**, *n* = 3 per genotype. All quantified data are presented as mean and SEM.
