# Supplementary material for: TRIB2 regulates normal and stress-induced thymocyte proliferation
Source: Cell Discov. 2016 Mar 15;2:15050–. doi: 10.1038/celldisc.2015.50 (PMC4860960; doi:10.1038/celldisc.2015.50)
Supplement: Supplementary Figure S5 [file celldisc201550-s5.pdf]

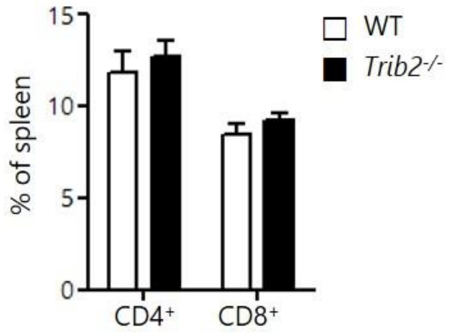

Figure S5. Distribution of $CD4^+$ and $CD8^+$ T cells in the spleen of WT (n = 4) and *Trib2*$^{-/-}$ (n = 5) mice at steady state. All quantified data are presented as mean and SEM.
