# Supplementary material for: TRIB2 regulates normal and stress-induced thymocyte proliferation
Source: Cell Discov. 2016 Mar 15;2:15050–. doi: 10.1038/celldisc.2015.50 (PMC4860960; doi:10.1038/celldisc.2015.50)
Supplement: Supplementary Figure S6 [file celldisc201550-s6.pdf]

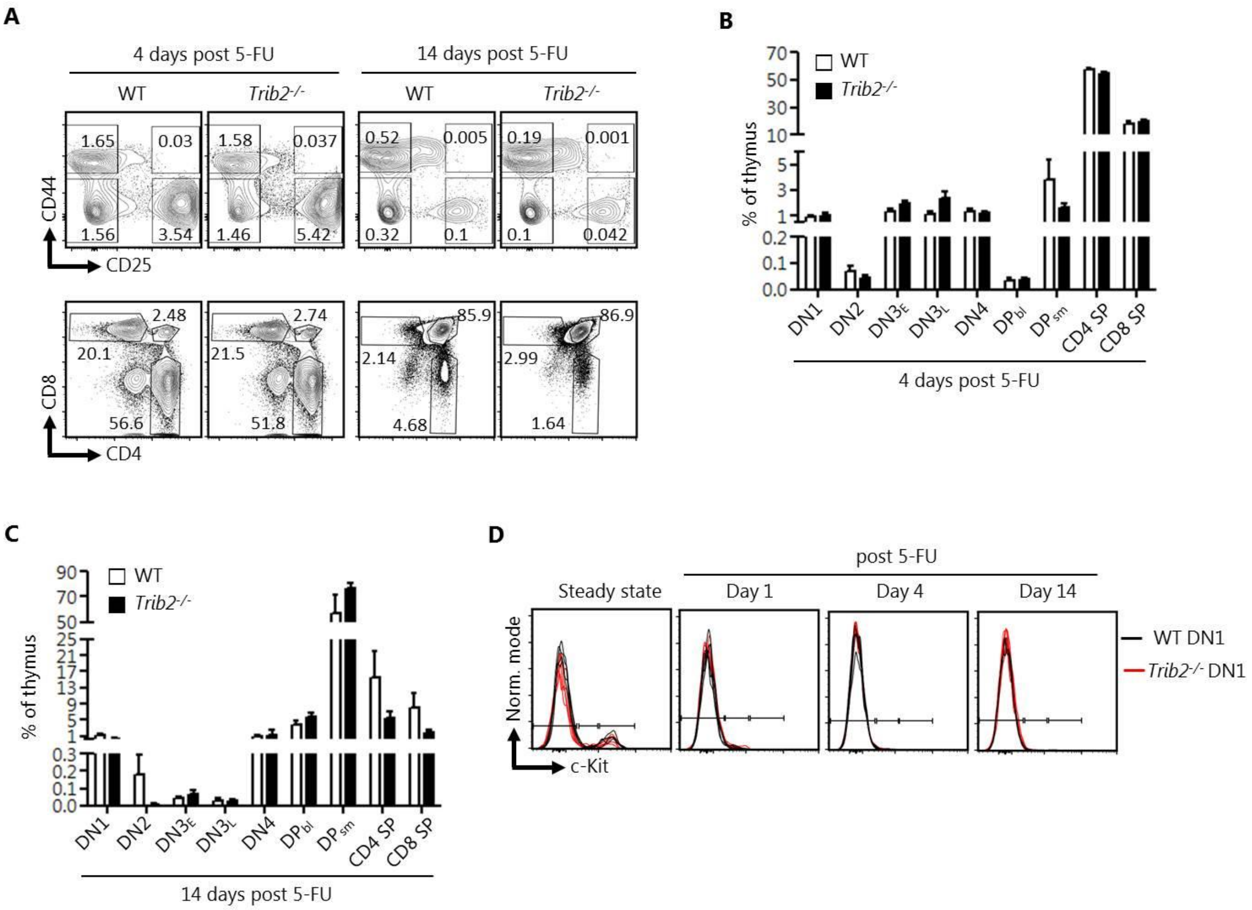

Figure S6. Distribution of thymic subsets after genotoxic insult. **(A)** A representative staining profile of WT and *Trib2*$^{-/-}$ thymus (*n* = 9 per genotype per studied time point) after 4 and 14 days of 5-FU treatment (250 mg/kg, i.p.) were shown here. The values indicated in the outlined areas are frequency of each thymic subset and graphed in **(B,C)**. **(D)** c-Kit expression of WT and *Trib2*$^{-/-}$DN1 thymocytes at steady state (*n* = 7-8 per genotype) and after 5-FU treatment (250 mg/kg, i.p.; *n* = 4-6 per genotype per studied time point) were shown in overlaid histograms. All quantified data are presented as mean and SEM.
