# Supplementary material for: TRIB2 regulates normal and stress-induced thymocyte proliferation
Source: Cell Discov. 2016 Mar 15;2:15050–. doi: 10.1038/celldisc.2015.50 (PMC4860960; doi:10.1038/celldisc.2015.50)
Supplement: Supplementary Figure S7 [file celldisc201550-s7.pdf]

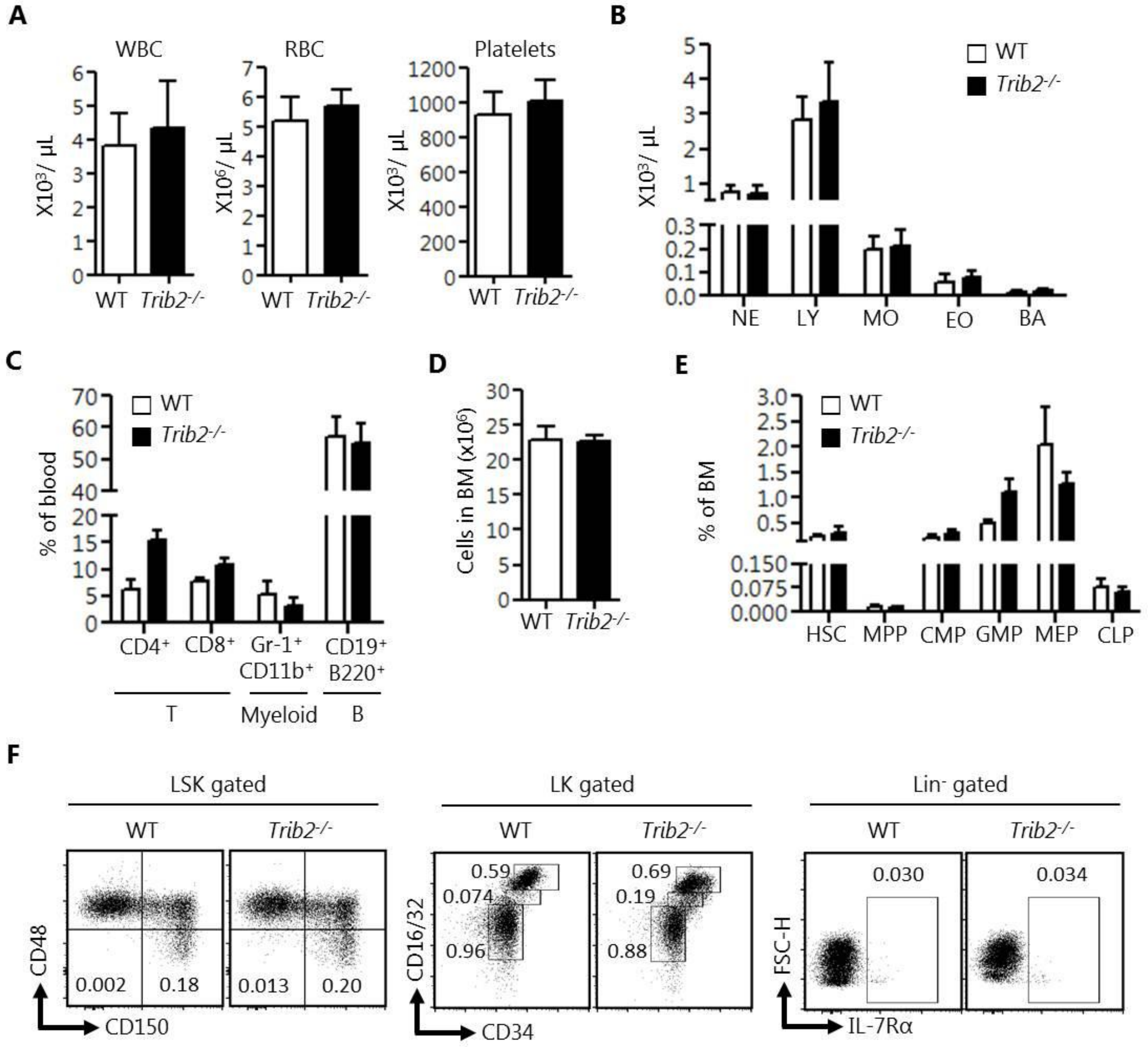

Figure S7. TRIB2 loss does not affect the restoration of hematopoietic system after 14 days of 5-FU treatment (250 mg/kg, i.p.). Complete blood counts **(A)** and WBC differential counts **(B)** of treated WT and *Trib2*$^{-/-}$ mice (*n* = 8-9 per genotype) were determined by hematology analyzer. **(C)** The distribution of mature myeloid, B and T cells in the blood of treated WT and *Trib2*$^{-/-}$ mice (*n* = 4-5 per genotype) were measured by flow cytometry. **(D)** BM cellularity was counted by trypan blue exclusion after RBC lysis of the cell suspension harvested from two pelvises, femurs and tibias. **(E)** Distribution of HSPC populations in BM of treated WT and *Trib2*$^{-/-}$ mice. **(F)** A

representative staining profile of the HSPC populations measured in WT and *Trib2*$^{-/-}$ mice is shown here. For **D-F**, *n* = 3 per genotype. All quantified data are presented as mean and SEM.
