# Supplementary material for: TRIB2 regulates normal and stress-induced thymocyte proliferation
Source: Cell Discov. 2016 Mar 15;2:15050–. doi: 10.1038/celldisc.2015.50 (PMC4860960; doi:10.1038/celldisc.2015.50)
Supplement: Supplementary Figure S8 [file celldisc201550-s8.pdf]

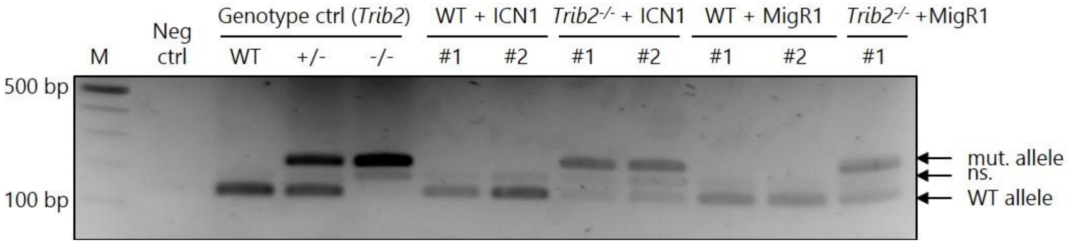

Figure S8. Mice in different groups of transplant experiments were verified by *Trib2* genotyping using splenic total genomic DNA. PCR analysis of representative samples from different groups is shown here. ns, non specific.
