# Supplementary material for: TRIB2 regulates normal and stress-induced thymocyte proliferation
Source: Cell Discov. 2016 Mar 15;2:15050–. doi: 10.1038/celldisc.2015.50 (PMC4860960; doi:10.1038/celldisc.2015.50)
Supplement: Supplementary Figure S9 [file celldisc201550-s9.pdf]

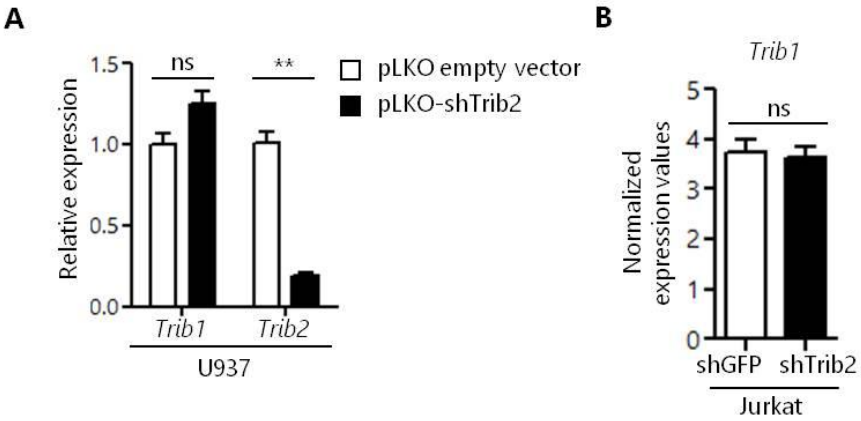

Figure S9. *Trib1* expression remained unchanged following *Trib2* knockdown. **(A)** Expression of *Trib1* and *Trib2* in GFP-sorted U937 cells was measured by quantitative RT-PCR after 48 hours of transduction with pLKO empty vector or pLKO-shTrib2. ns, not significant. **(B)** Normalized expression values of *Trib1* (average of 202241_at, 235641_at and 239818_x_at) in Jurkat cells following *Trib2* knockdown were derived from GSE66013 dataset [1]. For statistical analyses, unpaired Student's t-test was used for **A** and **B**. $^{**}P<0.01$, all quantified data are presented as mean and SEM.
