# Supplementary material for: TRIB2 regulates normal and stress-induced thymocyte proliferation
Source: Cell Discov. 2016 Mar 15;2:15050–. doi: 10.1038/celldisc.2015.50 (PMC4860960; doi:10.1038/celldisc.2015.50)
Supplement: Supplementary Table S1 [file celldisc201550-s10.pdf]

Table S1. Primers used for *Trib2* genotyping.

PCR primers	5'>3' sequence
Primer 1[A]	CACAATAGCGAGATATGGGAG
Primer 2	GCAATGCGACAAGTTCGGAG
Primer Neo3A	GCAGCGCATCGCCTTCTATC

[A] anneals to genome sequence deleted in the disrupted region of *Trib2* exon 1.
