# Supplementary material for: TRIB2 regulates normal and stress-induced thymocyte proliferation
Source: Cell Discov. 2016 Mar 15;2:15050–. doi: 10.1038/celldisc.2015.50 (PMC4860960; doi:10.1038/celldisc.2015.50)
Supplement: Supplementary Table S2 [file celldisc201550-s11.pdf]

Table S2. Primers used for quantitative qRT-PCR.

Gene	Forward primer (5' > 3')	Reverse primer (5' > 3')
Trib1	CTTCAAGCAGATTGTCTCCGC	CTAAGCTGGGTTCTCTCCTCC
Trib2	AGCCAGACTGTTCTACCAGA	GGCGTCTTCCAGGCTTTCCA
Abl	TGGAGATAACACTCTAAGCATAACTAAAGGT	GATGTAGTTGCTTGGGACCCA
B2m	TTGTCTTTCAGCAAGGACTGG	ATGCGGCATCTTCTAACCTCC
Enox2	GAGCTGGAGGGAACCTGATTT	CACTGGCACTACCAAACTGCA
Rnf20	GGTGTCTCTTCAACGGAGGAA	TAGTGAGGCATCATCAGTGGC
