# Supplementary material for: TRIB2 regulates normal and stress-induced thymocyte proliferation
Source: Cell Discov. 2016 Mar 15;2:15050–. doi: 10.1038/celldisc.2015.50 (PMC4860960; doi:10.1038/celldisc.2015.50)
Supplement: Supplementary Table S3 [file celldisc201550-s12.pdf]

Table S3. Primers used for detection of endogenous *Tcrb* rearrangements.

	Tcrb locus	5'>3' sequence	Annealing temperature (°C)
Forward primer	Dβ2	GTAGGCACCTGTGGGGAAGAAACT	58
	Vβ2	GGGTCACTGATACGGAGCTG	58
	Vβ4	GGACAATCAGACTGCCTCAAGT	58
	Vβ5.1	GTCCAACAGTTTGATGACTATCAC	56
	Vβ8	GATGACATCATCAGGTTTTGTC	56
	Vβ14	CTTCTACCTCTGTGCCTGGAGT	58
Reverse primer	Jβ2	TGAGAGCTGTCTCCTACTATCGATT	according to forward primer
