# Supplementary material for: TRIB2 regulates normal and stress-induced thymocyte proliferation
Source: Cell Discov. 2016 Mar 15;2:15050–. doi: 10.1038/celldisc.2015.50 (PMC4860960; doi:10.1038/celldisc.2015.50)
Supplement: Supplementary Method [file celldisc201550-s13.pdf]

Method S1. *Trib2* genotyping by PCR. WT and mutant *Trib2* alleles were detected and amplified by MangoMix PCR system (Bioline) using the primers listed in Table S1. PCR products were separated on 2.0% agarose gel, detected using SafeView Nucleic Acid Stain (NBS Biologicals), and imaged using ChemiDoc XRS system (Bio-Rad).

Method S2. Whole BM transplantation. $8x10^6$ BM cells were harvested from WT and *Trib2*$^{-/-}$ mice and were injected intravenously into lethally irradiated (2x4.25 grays fractionated doses were given with three hours apart) B6.SJL mice. Mice were monitored by periodic tail vein bleedings four weeks after transplantation. Mice from both groups were euthanized at 17 weeks after transplantation.

Method S3. Quantitative RT-PCR. High throughput quantitative RT-PCR analysis was performed on the Fluidigm 48.48 Dynamic Array Integrated Fluidic Circuits system (Biomark HD). Primer sequences are listed in Table S2. Specific target pre-amplification was carried out. Each target was measured in triplicate reactions. Expression levels of the target genes were normalized relative to the mean of the reference genes (Abl, *B2m*, *Enox2* and *Rnf20*). Relative mRNA levels were calculated using the 2-ΔΔCT method.

Supplementary references

1 Tan SH, Yam AW, Lawton LN *et al.* TRIB2 reinforces the oncogenic transcriptional program controlled by the TAL1 complex in T-cell acute lymphoblastic leukemia. *Leukemia* 2015.
